# Resolving inherited and *de novo* germline predisposing sequence variants by means of whole exome trio analyses in childhood hematological malignancies

**DOI:** 10.3389/fped.2022.1080347

**Published:** 2023-02-07

**Authors:** Triantafyllia Brozou, Layal Yasin, Danielle Brandes, Daniel Picard, Carolin Walter, Julian Varghese, Martin Dugas, Ute Fischer, Arndt Borkhardt, Oskar A. Haas

**Affiliations:** ^1^ Department of Pediatric Oncology, Hematology and Clinical Immunology, Medical Faculty, Heinrich Heine University, Düsseldorf, Germany; ^2^ Institute of Medical Informatics, University of Münster, Münster, Germany; ^3^ Insititute of Medical Informatics, Heidelberg University Hospital, Heidelberg, Germany; ^4^ St. Anna Children's Hospital, Pediatric Clinic, Medical University, Vienna, Austria

**Keywords:** pediatric hematological malignancies, trio-whole-exome sequencing, cancer predisposition syndrome, inheritance pattern, *de novo* mutations

## Abstract

Molecular screening tools have significantly eased the assessment of potential germline susceptibility factors that may underlie the development of pediatric malignancies. Most of the hitherto published studies utilize the comparative analyses of the respective patients' germline and tumor tissues for this purpose. Since this approach is not able to discriminate between *de novo* and inherited sequence variants, we performed whole exome trio analyses in a consecutive series of 131 children with various forms of hematologic malignancies and their parents. In total, we identified 458 *de novo* variants with a range from zero to 28 (median value = 3) per patient, although most of them (58%) had only up to three per exome. Overall, we identified *bona fide* cancer predisposing alterations in five of the investigated 131 (3.8%) patients. Three of them had *de novo* pathogenic lesions in the *SOS1*, *PTPN11* and *TP53* genes and two of them parentally inherited ones in the *STK11* and *PMS2* genes that are specific for a Peutz-Jeghers and a constitutional mismatch repair deficiency (CMMRD) syndrome, respectively. Notwithstanding that we did not identify a disease-specific alteration in the two cases with the highest number of *de novo* variants, one of them developed two almost synchronous malignancies: a myelodysplastic syndrome and successively within two months a cerebral astrocytoma. Moreover, we also found that the rate of *de novo* sequence variants in the offspring increased especially with the age of the father, but less so with that of the mother. We therefore conclude that trio analyses deliver an immediate overview about the inheritance pattern of the entire spectrum of sequence variants, which not only helps to securely identify the *de novo* or inherited nature of genuinely disease-related lesions, but also of all other less obvious variants that in one or the other way may eventually advance our understanding of the disease process.

## Introduction

Germline defects that predispose to the development of childhood malignancies comprise chromosome abnormalities, DNA sequence variants and epigenetic alterations (1–3). Decreasing costs and continuous improvements of genome-wide sequencing tools make it increasingly worthwhile to implement them into the routine diagnostic work-up of these diseases. The first retrospective study of 1,120 children and adolescents that aimed to determine the prevalence of such predisposing germline mutations found that 8.5% of the investigated patients were carriers of such disease-relevant changes (4). Similar results were then obtained in subsequent studies that reported overall frequencies between 7% and 14.6%. Moreover, they also noted that the probability to encounter them is considerably higher in patients with non-central nervous system solid tumors (16.7%) than in those with leukemias (4.4%) (1, 5–7). Pathogenic sequence variants that predispose to the manifestation of bone marrow failure and myelodysplastic syndromes, acute myeloid and lymphoblastic leukemias as well as lymphomas, in particular, affect primarily genes whose products contribute to the orderly development and differentiation of the respective tissues, which include various transcription factors, components of various signal transduction pathways and the immune system as well as constituents of the DNA maintenance and repair machinery (8–11). Most common in the context of lymphoblastic leukemias and lymphomas are thus mutations in the hematopoietic transcription factors *ETV6, IKZF1 and PAX5*, the tumor suppressor *TP53*, the DNA mismatch-repair genes *MLH1*, *MSH2*, *MSH6*, and *PMS2* as well as *ATM*, *NBN* and the *RAD51,* which are indispensable for the repair of double-strand breaks (8–10, 12–16). Amongst those whose dysfunction predisposes primarily to the development of myeloid malignancies are the transcription factors *GATA2* and *RUNX1,* members of the RAS signal transduction and ribosomal protein families, as well as components of the Fanconi and dyskeratosis congenita DNA maintenance and repair system (11, 15).

Given the overall large number of potentially predisposing genes together with the broad range of possible sequence variants that may be encountered even in already clinically recognizable syndromes, it is not surprising that genome-wide screening approaches, such as whole exome (WES) and whole genome sequencing, are nowadays already the preferred method to search for them.

Parent/child trio analyses are particularly suited to speed up the diagnostic process considerably because they can immediately discriminate *de novo* from inherited lesions and, especially in normal patients with unremarkable features, help to delineate rare causative genetic defects from innocuous variants, as well as well as advance the interpretation of those with an unknown significance.

Based on these arguments, we therefore introduced such WES trio analyses seven years ago in our laboratory and present herein the results that were obtained with this diagnostic approach in a consecutive and unselected cohort of 131 children with hematological malignancies.

## Material and methods

### Patients

In 2015, we initiated a prospective study in our Hematology and Clinical Immunology Department at the University Hospital Düsseldorf that aimed to assess the frequency and types of predisposing germline lesion in an unselected cohort of childhood malignancies by means of simultaneous comparative WES analyses of the patients and their parents (17). Enrolment required the informed consent from either the patients themselves, their parents, or their legal authorized representatives. The consent for the analyses of the parents' DNA was obtained after consultation with a pediatric oncologist who was certified in genetic counselling. The purpose of the study, the procedures to be followed, as well as the benefits and all foreseeable risks and adverse effects for the family were extensively discussed and explained in a clear language. All participants were offered sufficient time to consider, as well as the right to retract their consent at any time without need for further justification.

Once the consent was given, we systematically collected demographic and detailed medical data of the patient, based on already published screening questionnaire tools (18, 19). We focused on concomitant conditions suggestive of an underlying cancer predisposition syndrome and recorded the cancer history three generations back. This study was approved by the ethics committee of Heinrich Heine University, Düsseldorf, Germany (ethics vote number 4886R and study registration number 2014112933).

### Whole-exome sequencing

DNA was extracted from peripheral blood mononuclear cells (PBMCs) from the patients and their parents using the DNeasy Blood & Tissue kit (Qiagen). Whenever possible, we obtained the germline DNA from skin fibroblasts. In total, we sequenced in the patient cohort 62 fibroblast-derived and 69 PBMC-derived DNA samples. We tried to minimize the risk of malignant contamination by collecting blood in patients with leukemia or lymphoma with bone marrow involvement once patient has achieved remission. For WES we used the SureSelect Human All Exon V5 + UTR kit (Agilent). The library was paired end sequenced on an Illumina HiSeq2500 (2 × 100 bp) or NextSeq550 (2 × 150 bp) sequencer with an average on target coverage of ≥80x. Details of the bioinformatic processing and analyses of the WES data are provided in the Supplementary Methods. We validated all pathogenic and likely pathogenic variants by PCR-based Sanger sequencing.

### Classification of the identified sequence variants

We applied two different published tools for automated variant interpretation. The first was based on rules that were adapted from the CharGer tool (Supplementary Table S1) (20, 21) and the second one was the CPSR pipeline (22). We initially concentrated on a preselected panel of 295 genes known to be associated with childhood-onset cancer predisposition syndromes (containing tumor suppressor genes, genes involved in cell cycle arrest and DNA damage repair, RASopathy genes) and we also mined the literature and assessed the gene panels used in previous studies. The genes were divided into three different categories based on their level of evidence being associated to cancer predisposition. Recognized variants were then classified according to their likely effects as pathogenic, likely pathogenic, benign, likely benign or variant of unknown significance (VUS). After the initial classification according to CharGer and CPSR we proceeded to manually revise all the detected variants regardless of their *in silico* prediction status (benign, VUS, pathogenic), taking in consideration the existing literature and the patients' phenotypes as well as information that derived from their three-generation pedigree. The validity and relevance of these results were then finally evaluated and interpreted by an interdisciplinary team that consisted of basic researchers and senior pediatric oncologists. Although their assessment was based on the ACMG standards and guidelines (23), unclear lesions that might originally have been classified as pathogenic or likely pathogenic by the interpretation tools could be revised as a VUS.

## Results

### Description of an unselected cohort of children with hematological malignancies

The cohort reported herein consists of 131 children and adolescents up to 19 years of age that were diagnosed with various types of hematologic malignancies (Table 1). Sixty-nine patients had a B-cell precursor acute lymphoblastic leukemia (BCP-ALL), 13 a T-ALL, 15 an acute myeloid leukemia (AML) and one each a chronic myeloid leukemia (CML) and Burkitt leukemia, respectively. In addition, there were 31 patients with either a Hodgkin (*n* = 15) or non-Hodgkin lymphomas (*n* = 16) that comprised Burkitt, diffuse large B-cell (DLBCL), T-cell and anaplastic large cell lymphoma (ALCL) (Table 1). One patient in our cohort presented with a myelodysplastic syndrome with refractory cytopenia and developed within two months after diagnosis also an astrocytoma. The male to female ratio of the patients was 1.5:1, and their median age at disease onset was 5.4 years (range 0.1–18.5). Matching to the true distribution of pediatric hematological malignancies in childhood we observed a high proportion of BCP-ALL patients, whereas only a minority of the patients presented with AML/MDS in our cohort, a cancer subtype associated with higher rate of underlying germline defects. The clinical features of the patients are summarized in the Supplementary Table S2.

**Table 1:** Clinical characteristics of the 131 patients reported in this study.

	Number of cases (Frequency)
Sex	
Male	80 (61%)
Female	51 (38.9%)
Age at diagnosis	
0–5 years	72 (54.9%)
6–10 years	23 (17.5%)
11–15 years	25 (19%)
16–18 years	11 (8.3%)
Diagnosis	
Leukemia	99 (75.5%)
B-cell acute lymphoblastic leukemia	69
T-cell acute lymphoblastic leukemia	13
Acute myeloid leukemia	15
Chronic myeloid leukemia	1
Burkitt leukemia	1
Lymphoma	31 (23.6%)
Hodgkin lymphoma	15
Burkitt lymphoma	6
T-cell lymphoma	4
Diffuse large B-cell lymphoma	4
Anaplastic large cell lymphoma	2
Other hematopoietic malignancies	(0.76%)
Myelodysplastic syndrome	1

### Pathogenic/likely pathogenic germline variants in cancer predisposition genes

We primarily focused our analyses on a virtual panel of 295 candidate genes (Supplementary Table S3) and classified the respective sequence changes according to the above defined criteria. Five of the 131 examined patients (3.8%) had a pathogenic or likely pathogenic sequence variant. Three of these occurred *de novo* and two were inherited.

Two of the patients with *de novo* mutations had Noonan syndrome. In patient P-31, who developed a high-hyperdiploid BCP-ALL at the age of 2.5 years, it was caused by a *PTPN11* (p.Asn308Asp) mutation and in Patient P-120, who developed *ETV6::RUNX1*-positive BCP-ALL when he was 1.7 years old, it resulted from an already previously reported Noonan-associated *SOS1* (p.Phe868Leu) mutation (24, 25). The third patient P-48, who was diagnosed with a BCP-ALL leukemia at the age of 9.7 years, had a Li-Fraumeni syndrome-associated *de novo* stop/gain *TP53* (p.Arg196*) mutation that had already previously been reported by us (7). Matching to the diagnosis of a Li-Fraumeni syndrome the patient had a leukemia with a low hypodiploid karyotype with 37 chromosomes. The prompt diagnosis of a Li-Fraumeni syndrome had a significant impact on the clinical decision-making, as we preferred a chemotherapy-based conditioning regimen prior to bone marrow transplantation for this patient and omitted irradiation.

Patient P-67, whose parents were first degree cousins, was diagnosed with a T-cell lymphoma at the age of 3.5 years. One of his siblings developed a medulloblastoma when he was three years old and died soon after because of progressing disease. The maternal grandfather had a colon adenocarcinoma. Although we suspected a causative constitutional mismatch repair gene defect (CMMRD), WES did not reveal any suspicious variant in the respective genes. We therefore Sanger sequenced them and thereby uncovered a homozygous *p*.His845Asp mutation in the *PMS2* gene. These variants were originally not detected, because they are in a highly homologous region that was insufficiently covered by WES. Notwithstanding the fact that the *in-silico* analysis interpreted them as VUS, their specific nature and the highly remarkable family history prompted us to confidently re-classify them as pathogenic. Based on this decision, we counselled the family accordingly and offered them to participate in a CMMRD-specific clinical surveillance program.

Patient *P*-117, who presented with a T-ALL when he was three years old, had a maternally transmitted Peutz-Jeghers syndrome-related stop/gain *STK11* (p.Tyr60Ter) mutation, that was identified through sequencing of fibroblast-derived DNA. He as well as his mother and maternal grandfather, who were also carriers, had the typical multiple hyperpigmented macules on the lips and oral mucosa. Individuals with pathogenic germline *STK11* variants have a lifelong elevated risk to acquire various types of malignancies, such as gastrointestinal, pancreatic, testicular, cervical and breast cancer, that mainly manifest themselves during adulthood (26–28). However, to the best of our knowledge, no leukemia cases have so far been reported in this condition and somatic *STK11* P280S mutations were previously reported in only two children with ALL: one child with B-ALL and one with Philadelphia chromosome–like acute lymphoblastic leukemia (Ph-like ALL) (29, 30).

The patients detected with a cancer predisposition syndrome were consecutively referred to the Human Genetics Department for further genetic counseling with their relatives and enrolled in appropriate cancer surveillance programs.

### Inherited vs. *de novo* sequence variants

**Figure 1:**
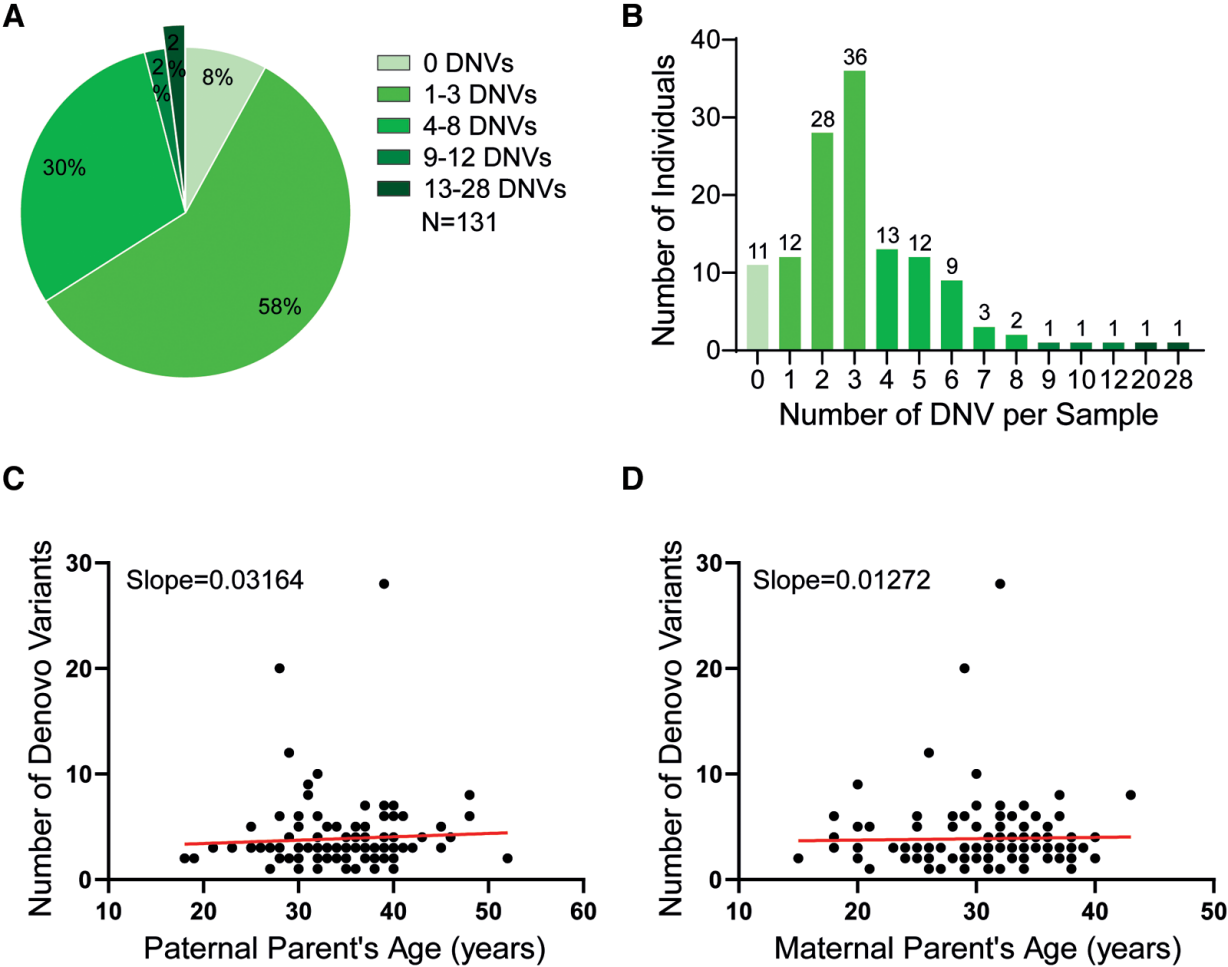
(**A**) The pie chart depicts the number distribution of the *de novo* variants (DNVs) that were identified in our cohort (**B**) The bar plot depicts the number of *de novo* variants in the exomes per sample. (**C**) The dot plot shows the correlation between the paternal age and the *de novo* variants (DNV) frequency in his offspring. Each dot defines the numbers of DNV (*y*-axis) in relation to the age of the respective fathers (*x*-axis) when their children were born. Linear regression analysis defines a best-fit line with an intercept of 2.7854 and a slope of 0.03164 that verifies a positive correlation between these two parameters. (**D**) The dot plot shows the correlation between the maternal age and the DNV frequency in her offspring. Each dot defines the numbers of DNV (*y*-axis) in relation to the age of the respective mothers (*x*-axis) when their children were born. Linear regression analysis defines a best-fit line with an intercept of 3.49049 and slope of 0.01272 that verifies a positive correlation between these two parameters.

Our WES analyses uncovered overall 117,178 non-synonymous inherited and *de novo* sequence changes in 15,490 genes, which comprised frameshift indels, in frame indels, missense, stop/gain, start/lost, stop/loss, and splice sites region (±3–8bpIn) ones. Virtually all of them (116,720/117,178; 99.6%) were inherited from either one of the parents and only 458 (0.4%) of them occurred *de novo*. The rate of exonic *de novo* sequence variants (DSV) in our study, both single-nucleotide variants (SNVs) and indels is estimated to be 9 × 10^−8^ per base pair per generation. As expected, missense variants was the most common type of mutations occurring *de novo* with a percentage of 51% (236/458). An overview of the various types of *de novo* variants is illustrated in Supplementary Table S4. Eleven (8.3%) patients had none, 58% one to three (Figure 1A), another 30% between four and eight and only five more than nine such *de novo* variants in the exonic part of the genome that was covered by our analyses. The number of *de novo* variants ranged from zero to 28 per exome (Figure 1B). Two patients, P-99 and P-118, had an exceptionally high number of 20 and 28 *de novo* variants, yet no obvious disease-relevant pathogenic ones. Still, the clinical course of patient P-118 is especially noteworthy, because he developed a myelodysplastic syndrome when he was 13.4 years old and already two months later also a cerebral astrocytoma.

### Association of *de novo* variants to parental age

The median paternal and maternal age at childbirth was 35 and 31 years, respectively, with an age range from 15 to 43 years for the mothers and 18–52 years for the fathers. Linear regression analyses revealed that the increasing numbers of *de novo* variants in the investigated children correlated with the age of their respective parents and especially with that of their fathers (Figures 1C,D).

**Figure 2:**
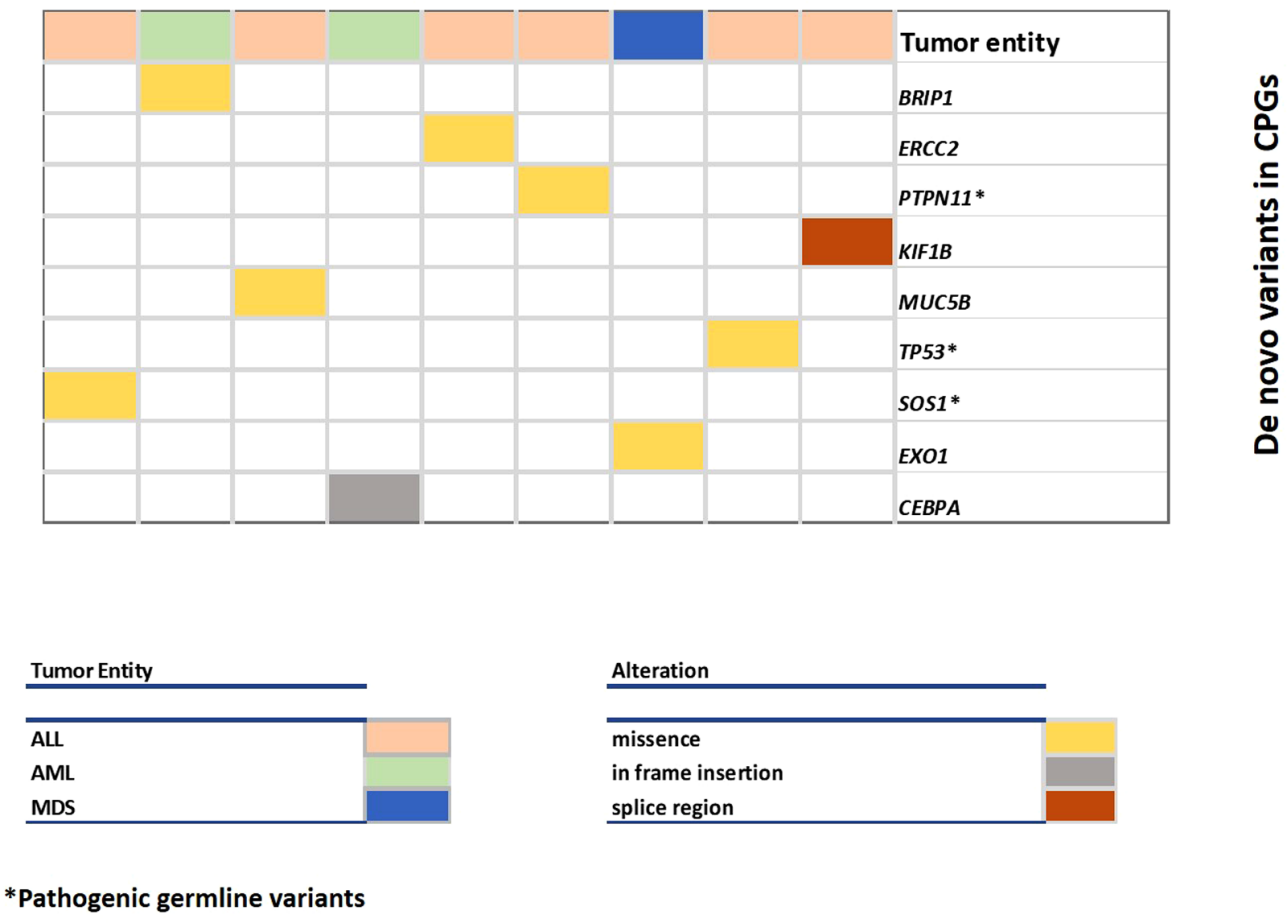
Overview of DNVs in cancer predisposition genes. Each column represents a case and each row represents a gene. The color of the columns indicates the type of malignancy (leukemia, myelodysplastic syndrome) and the color of the row indicates the type of alteration.

Only nine (6.9%) patients had a *de novo* mutation in one of the 295 candidate genes, three of which were pathogenic (Table 2, Figure 2). The ratio of nine *de novo* vs. 1,609 transmitted variants was only slightly higher (0.55%) in these genes than in the remaining exome (449 *de novo* vs. 115,111 transmitted variants; 0.39%), a difference that is statistically not significant (Fischer exact test 0.3086).

**Table 2:** Overview of the pathogenic/likely pathogenic variants that were identified in the five syndromic cases.

Case	Gene/transcript	Genomic location in bp	Consequence on cDNA level	Consequence on protein level	Zygosity	Inheritance	Clinical signs	Associated syndrome	Diagnosis
P-31^a^	*PTPN11* ENST00000635625	chr12:112915523 A > G	c.922A > G	p.Asn308Asp	Heterozygous	*de novo*	2	Noonan	BCP-ALL
P-120	*SOS1* ENST00000426016	chr2:39022774T > C	c.1654A > G	p.Phe868Leu	Heterozygous	*de novo*	1	Noonan	BCP-ALL
P-48^a^	*TP53* ENST00000269305	chr17:7674945 G > A	c.586C > T	p.Arg196*	Heterozygous	*de novo*	3	Li-Fraumeni	BCP-ALL
P-67^a^	*PMS2*^b^ ENST00000265849	chr7:5973455G > C	c.2533C > G	p.His845Asp	Homozygous	Inherited	2	CMMRD	T-cell Lymphoma
P-117	*STK11* ENST00000326873	chr19:1207091-1207092 T > TA	c.179dup	p.Tyr60Ter	Heterozygous	Inherited	1	Peutz-Jeghers	T-ALL

Clinical signs: Number of clinical signs indicating the presence of the respective syndrome; CMMRD, Constitutional mismatch repair deficiency syndrome.

* stands for stop gain.

^a^ Previously reported by Wagener *et* al.

^b^ *PMS2* variant was identified through Sanger sequencing.

## Discussion

Our WES trio analyses of 131 consecutively diagnosed patients with hematologic malignancies uncovered a responsible predisposing *de novo* germline defect in three and an inherited one in two of them. Remarkably, these were all patients with a clinically recognizable syndrome that directed us already to the most likely involved genes. Apart from that, we did not discover any predisposing germline alterations in phenotypically inconspicuous cases in this series. Nevertheless, these observations are in good agreement with previous studies that reported a similar overall incidence in children with hematologic malignancies (4.4%), which is considerably lower than the one reported in those with central nervous system (8.6%) and non-central nervous system tumors (16.7%) (1, 5–7).

Owing to the decreasing costs, WES analyses is increasingly being used over Sanger and targeted sequencing approaches to screen for potential germline predisposing factors in patients with malignancies. The fact that we identified in our series only disease relevant germline alterations in phenotypically already recognizable syndromic but none in phenotypically inconspicuous patients, could leave the impression that rather than performing trio analyses instantly at disease onset, it would be more appropriate and cost efficient to simply ascertain the disease-relevant alteration first in the patient herself before one turns to carrier screening of blood related family members with simpler PCR-based methods. Yet, even in such constellations the advantage of trio analyses is not only that one will instantaneously identify the *bona fide* disease-relevant alterations in the respective families, but at the same time also obtain a complete overview about the entire spectrum of *de novo* occurring and inherited sequence changes that, in less clear situations, may help to better asses the biological and clinical relevance of the questionable ones. Examples are rare variants that are not necessarily viewed as pathogenic, but which nevertheless can, under specific circumstances, contribute to disease development, as has for instance been shown in the bi-parental inheritance of rare variants in two different genes (31).

The application of family-based WES offers unique insights in the detection of cancer predisposition syndromes in children and their relatives. It is the optimal method for detecting homozygous, compound heterozygous, digenic and *de novo* pathogenic variants and subsequently predict the recurrence risk in the affected family. Additionally, there is a plethora of children and families harboring strong clinical signs indicative of a CPS and lacking a convincing genetic explanation. This realization suggests that our horizon regarding cancer predisposition genes and variants is still limited. Under this scope, extended comprehensive TRIO-WES analysis is necessary in order to gain further knowledge on tumorigenesis. Especially in the case of variants of unknown significance, the inheritance pattern provided from the TRIO-WES in combination with the clinical data of the respective parental lineage proves to be a valuable and unreplaceable tool in the manual evaluation and final classification of the detected variants. Interestingly, the affected families express a very high interest in participating in extended WES-studies, which reaches up to 90% (32).

Nevertheless, the broad application of family-based WES sequencing in the clinical setting of pediatric oncology has noteworthy limitations. The consent for the genetic analysis lies on the parents or legal representatives and thus the children are deprived from the right to decide autonomously when reaching adulthood. Additionally, the accurate evaluation of the WES results presents a challenge, regarding the plethora and complexity of the findings that arouse. The interpretation of rare variants and variants of unknown significance requires caution and we must be aware that investigators may often assess variants with ambiguity given the rarity of the respective diseases, as well as the limited preexisting information and experience. Thus, in many cases due to our restricted ability to assess the pathogenicity of detected variants, further functional validation and time-consuming work-up are required. The identification and disclosure of non-cancer related secondary findings can be an additional psychological burden for the families, although in case of actionable genes, such as those recommended in the American College of Medical Genetics and Genomics (ACMG) SF v3.0 list, the prompt detection of pathogenic variants can lead to the implementation of life-saving prevention measures (17).

Since virtually all *de novo* sequence variants are already created in the germ cells of their respective parents, they also provide valuable insights into the mutagenic processes that take place in these tissues. The number and genomic location of such DSV is determined by the parents' sex and their age at conception as well as by the sequence and functional features of the regions in which they take place (33). Given that the estimated rate of such DSV lies between 1 × 10^−8^ and 1.2 × 10^−8^ per base pair per generation, newborns carry an average of 60 (between 30 and 100) DSV (34–37). The rate of these mutations increases in the father's germ cells by one per every additional year, which is consistent with a 16.5-years doubling rate, and in the mother by four per every additional year. The main reasons for these sex- and age-dependent mutation rates are that spermatogonia are continuously produced during the entire life with an average of 23 mitosis per year, whereas oocytes do not divide anymore after birth.

Except for two cases with an exceptionally high number of *de novo* sequence variants in which we were unable to define a genuinely disease predisposing one, the rate of all other patients was within the normal range. To which extent such apparently innocuous inherited and *de novo* variants, might still influence the likelihood of disease manifestation and its further development by impacting the mutational processes in the respective somatic cells, remains to be investigated (11, 38, 39). Although this information might at present be primarily of scientific interest, it may certainly also become of practical and clinical relevance in the future.

## Data Availability

The datasets presented in this study can be found in online repositories. The name of the repository is EGA and accession numbers are listed here: EGAN00004217198, EGAN00004217199, EGAN00004217200, and EGAN00004217197.
