# Supplementary material for: Resolving inherited and de novo germline predisposing sequence variants by means of whole exome trio analyses in childhood hematological malignancies
Source: Front Pediatr. 2023 Feb 7;10:1080347. doi: 10.3389/fped.2022.1080347 (PMC9941195; doi:10.3389/fped.2022.1080347)
Supplement: Supplementary file 1 [file Table1.docx]

**Supplementary Methods**

**Bioinformatic analysis of whole-exome sequencing data**

*Variant calling*

Reads were extracted using bcl2Fastq v2.19.0 and adapters were trimmed using trimmomatic v0.39. Afterwards, duplicate reads were marked using Picard tools 2.0.1 (http://broadinstitute.github.io/picard/). Alignment to human reference genome GRCh38 (genbank assembly GCA_000001405.28) was done using BWA-MEM v0.7.17 and Samtools v2.23.8. To evaluate the coverage of the WES data, we applied bedtools v2.16. and bamstats04 (http://dx.doi.org/10.6084/m9.figshare.1425030). We confirmed the familial relationship of the trio-WES data applying Peddy 0.4.6. Single nucleotide variants (SNVs) and insertion/deletions (indels) were called using GATK v4.1.4.1 (filter settings for SNVs: QD<2.0, QUAL><0.0, SOR>2.25, MQ <40.0 and for indels: QD<2.0, QUAL<30.0, SOR>2.75, MQ <55.0) and VarScan2 v2.3.9 (filter settings: min-coverage 10, min var-freq 0.20, p-value 0.05, adj-var-freq 0.05, adj-p-value 0.15). Both variant callers were applied in the trio mode, allowing for differentiation between transmitted and de novovariants. In addition, platypus v0.8.1 was used to call indels (default filter setting). All resulting SNVs from GATK and VarScan2 were combined into one SNV dataset whereby indels called by at least 2 of the 3 callers (GATK, VarScan2, Platypus) and all additional unique indels from GATK were considered for further analysis. Taken together, our final variant data set comprised of single nucleotide variants and small insertions/deletions.

*Variant filtering*

We excluded variants (i) with a variant allele frequency of <10% in the child, (ii) with a reported minor allele frequency (MAF) of >1% according to the gnomAD non-cancer population and (iii) which occurred in ≥5 % of cases in our cohort. The list of variants was further reduced to 295 genes, which can be separated into three different categories. Category one encompassed 151 genes described by Zhang et al. to be either autosomal dominant or autosomal recessive inherited, and to be tumor suppressor genes. In addition, genes described to be associated with rasopathies were included.

Category two encompassed 117 genes not included in category 1 but (i) which were described by different germline studies to be associated with cancer predisposition, as well as (ii) different kinase genes described to be associated with cancer predisposition. Category three included 27 genes which have been described with germline variants most likely predisposing to hematological malignancies and which were not already included in group 1 and 2. Refer to table S2 for a complete overview of the genes analyzed.

*Variant annotation*

Functional annotation of variants was done using Ensembl Variant Effect Predictor v98.3. For in silico prediction of the effect of the variants, SIFT, Polyphen and CADD were applied. The COSMIC database (downloaded 25.03.2019 https.//cancer.sanger.ac.uk/cosmic/download) was used to identify variants located in somatic mutational hotspots. In addition, we used the ClinVar database (download 02/12/2019), the IARC TP53 germline database and the LOVD database for MSH2, MSH6, APC and NF1 in order to identify previously reported pathogenic variants. In addition, we used the dbNSFP 3.5 plugin to annotate the conservation scores based on GERP++ and phastCons100way_vertebrate. For in silico prediction of the effect of splice site variants, we applied the dbscSNV v1.1 plugin for VEP which annotated the ada and rf-scores to the splice variants. Furthermore, we applied the Human Splicing Finder 3.1 to classify the splicing effect of splice region variants (+/-3-8 bp).
